# Supplementary material for: A new protocol for multispecies bacterial infections in zebrafish and their monitoring through automated image analysis
Source: PLoS One. 2024 Aug 8;19(8):e0304827. doi: 10.1371/journal.pone.0304827 (PMC11309447; doi:10.1371/journal.pone.0304827)
Supplement: S1 File — Also available on protocols.io. (PDF) [file pone.0304827.s001.pdf]

A new protocol for multispecies bacterial infections in zebrafish and their monitoring through automated image analysis

RESERVED DOI:
10.17504/protocols.io.rm7vzjybxlx1/v1

Désirée A. Schmitz[1,2], Tobias Wechsler[1], Hongwei Bran Li[1,3], Bjoern H. Menze[1], Rolf Kümmerli[1]

[1]Department of Quantitative Biomedicine, University of Zurich, Zurich, Switzerland;
[2]Department of Microbiology, Harvard Medical School, Boston, Massachusetts, USA;
[3]Athinoula A. Martinos Center for Biomedical Imaging, Massachusetts General Hospital, Harvard Medical School, Boston, Massachusetts, USA

Desiree Schmitz
Harvard Medical School

Collection Info: Désirée A. Schmitz, Tobias Wechsler, Hongwei Bran Li, Bjoern H. Menze, Rolf Kümmerli . A new protocol for multispecies bacterial infections in zebrafish and their monitoring through automated image analysis. **protocols.io**
https://protocols.io/view/a-new-protocol-for-multispecies-bacterial-infectio-dc462yze

Created: April 16, 2024

Last Modified: May 01, 2024

Collection Integer ID: 99198

Funders Acknowledgement:
Swiss National Science Foundation
Grant ID: 310030_212266
Swiss National Science Foundation
Grant ID: 31003A_182499

Abstract

The zebrafish *Danio rerio* has become a popular model host to explore disease pathology caused by infectious agents. A main advantage is its transparency at an early age, which enables live imaging of infection dynamics. While multispecies infections are common in patients, the zebrafish model is rarely used to study them, although the model would be ideal for investigating pathogen-pathogen and pathogenhost interactions. This may be due to the absence of an established multispecies infection protocol for a defined organ and the lack of suitable image analysis pipelines for automated image processing. To address these issues, we developed a protocol for establishing and tracking single and multispecies bacterial infections in the inner ear structure (otic vesicle) of the zebrafish by imaging. Subsequently, we generated an image analysis pipeline that involved deep learning for the automated segmentation of the otic vesicle, and scripts for quantifying pathogen frequencies through fluorescence intensity measures. We used *Pseudomonas aeruginosa, Acinetobacter baumannii, and Klebsiella pneumoniae*, three of the difficult-to-treat ESKAPE pathogens, to show that our infection protocol and image analysis pipeline work both for single pathogens and pairwise pathogen combinations. Thus, our protocols provide a comprehensive toolbox for studying single and multispecies infections in real-time in zebrafish.

Files

SEARCH

Protocol

NAME

Protocol (A): Zebrafish infections into the otic vesicle (2 dpf)

VERSION DC452YY6

CREATED BY

Desiree Schmitz
Harvard Medical School

OPEN →

Protocol

NAME

Protocol (B): Zebrafish embedding and imaging (3 dpf)

VERSION DC472YZN

CREATED BY

Desiree Schmitz
Harvard Medical School

OPEN →

Protocol

NAME

Protocol (C): Automated segmentation of the otic vesicle and image analysis

VERSION DC482YZW

CREATED BY

Desiree Schmitz
Harvard Medical School

OPEN →
