# Supplementary material for: A new protocol for multispecies bacterial infections in zebrafish and their monitoring through automated image analysis
Source: PLoS One. 2024 Aug 8;19(8):e0304827. doi: 10.1371/journal.pone.0304827 (PMC11309447; doi:10.1371/journal.pone.0304827)
Supplement: S2 File — Also available on protocols.io. (PDF) [file pone.0304827.s002.pdf]

protocols.io Part of SPRINGER NATURE

Protocol (A): Zebrafish infections into the otic vesicle (2 dpf)

In 1 collection

RESERVED DOI:
10.17504/protocols.io.j8nlk8kwwl5r/v1

Désirée A. Schmitz[1,2], Tobias Wechsler[1], Hongwei Bran Li[1,3], Bjoern H. Menze[1], Rolf Kümmerli[1]

[1]Department of Quantitative Biomedicine, University of Zurich, Zurich, Switzerland;
[2]Department of Microbiology, Harvard Medical School, Boston, Massachusetts, USA;
[3]Athinoula A. Martinos Center for Biomedical Imaging, Massachusetts General Hospital, Harvard Medical School, Boston, Massachusetts, USA

Desiree Schmitz
Harvard Medical School

Protocol Info: Désirée A. Schmitz, Tobias Wechsler, Hongwei Bran Li, Bjoern H. Menze, Rolf Kümmerli . Protocol (A): Zebrafish infections into the otic vesicle (2 dpf). **protocols.io** **https://protocols.io/view/protocol-a-zebrafish-infections-into-the-otic-vesi-dc452yy6**

Created: April 16, 2024

Last Modified: May 01, 2024

Protocol Integer ID: 99197

Abstract

This protocol details the zebrafish infections into the otic vesicle.

Guidelines

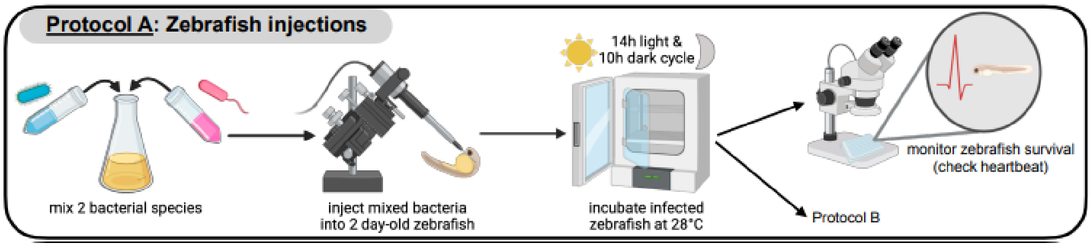

Table SP1. Media preparation of E3 zebrafish water (60x stock and 1x), pronase for dechorionation, PTU to avoid pigmentation, tricaine for anesthetizing.

A	B	C
Solution	Amount	Chemical
E3 60x stock		
	34.4 g	NaCl
	1.52 g	KCl
	2.9 g	CaCl * 2H20
	9.8 g	MgSO4 * 2H2O
		Fill up to 2000 mL with milliQ water
		Stir and autoclave (1 L suffices for 60 L 1x E3)
E3 1x		
	165 mL	60x E3
	10 L	milliQ water
Pronase		
Aqueous solution stable for 1 y at -20°C	5 g	Pronase (from Roche)
Shelf life: as indicated	167 mL	milliQ water
1. Mix 5 g Pronase with 167 mL with stirrer **under hood** until pronase is dissolved. 2. Store at -20°C in 1 mL aliqouts.		
PTU		N-Phenylthiourea (e.g. P7629-10 g)
Store powder in toxic material area (Shelf life: indefinite)	304 mg	PTU
Option1: **10x**		#ERROR

A	B	C
		Stir overnight in fumehood (toxic!), cover in aluminum foil
Option2: **2500x** first dissolve in DMSO & store aliquots at 4°C	750 mg	PTU
	10 mL DMSO	Stir overnight, cover in aluminum foil
		=2'500X STOCK → ADD 400 ML TO 1 L E3 (0.003% PTU)
**Tricaine/Mesab**		
(= 4000 mg/L)	4g	Tricaine/Mesab
		Fill up with milliQ water to 1000 mL
		Adjust pH at 7.0
		Store at -20°C

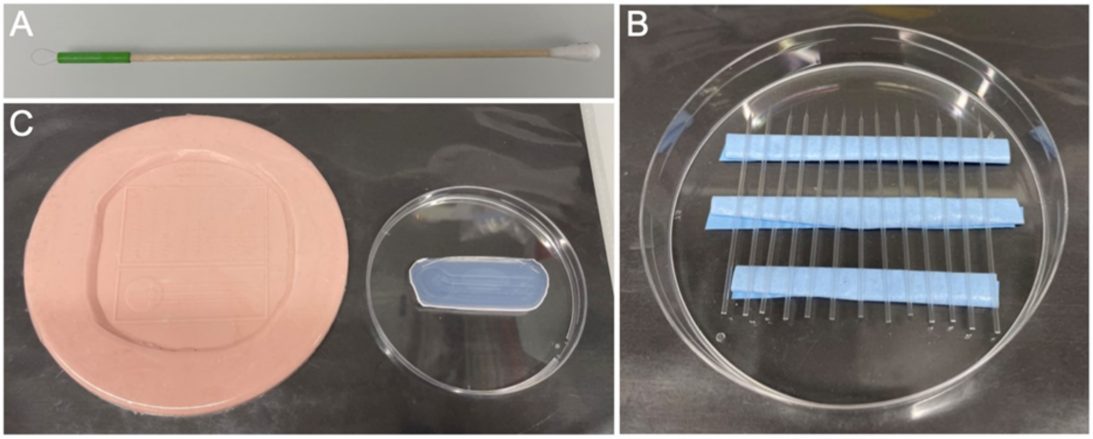

Figure SP1. **Material that needs to be prepared prior to injections.**
(A) Hair loop manipulator. (B) Needles. (C) Rubber mold (left) and agarose mold made with it (right).

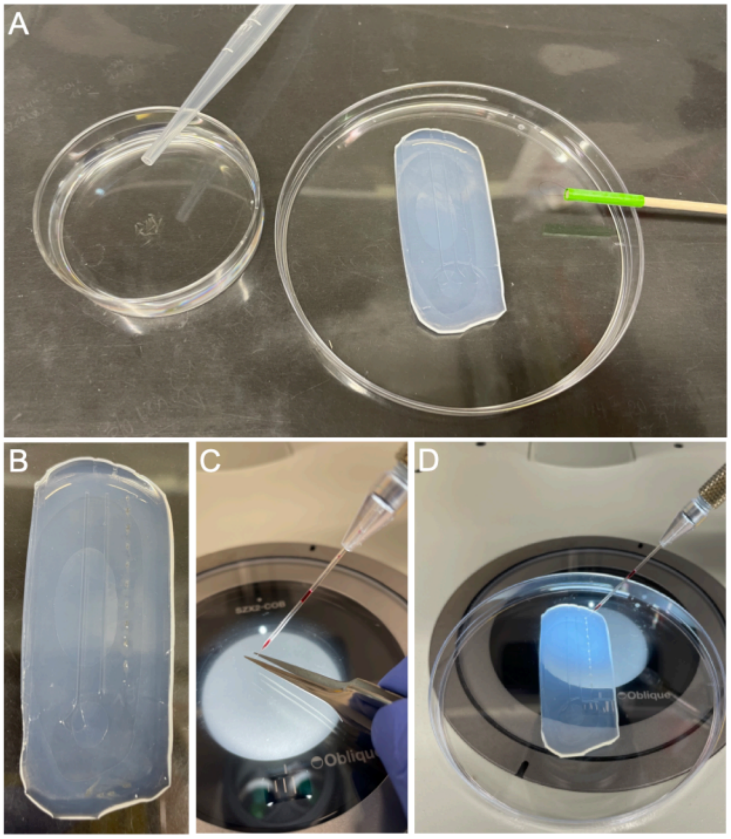

Figure SP2. Steps illustrating the injection protocol. (A) On the left are 2-days-post-fertilization zebrafish in a small petri dish containing an anesthetizing solution (tricaine). A Pasteur pipette can be seen on top for moving the zebrafish from the petri dish onto the agarose mold. On the right is an agarose mold (placed on an inverted lid of a petri dish) and a hair loop manipulator. (B) An agarose mold with zebrafish aligned in the rightmost channel.(C) Breaking off the needle with fine tweezers using a stereomicroscope. The needle is held by a micromanipulator. (D) Injecting zebrafish using a stereomicroscope and a micromanipulator holding a glass needle.

Materials

- All media were purchased from Sigma Aldrich, Switzerland unless indicated otherwise.
- All experiments in this study were conducted using lysogeny broth (LB).

Equipment:
For this protocol, you will need the following equipment:
- An incubator set to 37°C at 170 rpm with aeration (e.g., Infors, Multitron Standard Shaker)
- A spectrophotometer to measure optical density at a wavelength of 600 nm
- A centrifuge similar to MPW Med. Instruments, MPW-352R
- A stereomicroscope with a reticle and ruler plate in one ocular, e.g. Olympus SZX7 (final magnification of 56X)
- Micropipette puller device, for example, Sutter Instrument Co., Model Flaming/Brown P-87 with the following settings: air pressure=500, heat=609, pull=200, velocity=100, time=50
- A micromanipulator, e.g. Märzhäuser Wetzlar, MM33
- An injection pump similar to the Eppendorf FemtoJet 4i
- A temperature- and light-controlled incubator set to 28°C and a 14h light- and 10h dark-cycle, e.g., Memmert IPP110ecoPlus
- A negative rubber mold of a microstructured surface array from Ellet & Irimia (Ellett F, Irimia D. Microstructured Surface Arrays for Injection of Zebrafish Larvae. Zebrafish 2017;14:140–5. https://doi.org/10.1089/zeb.2016.1402). Their device is available for purchase at BioMEMS Core at the Massachusetts General Hospital (https://researchcores.partners.org/biomem/about).

Table SP1. Media preparation of E3 zebrafish water (60x stock and 1x), pronase for dechorionation, PTU to avoid pigmentation, tricaine for anesthetizing.

A	B	C
Solution	Amount	Chemical
**E3 60x stock**		
	34.4 g	NaCl
	1.52 g	KCl
	2.9 g	CaCl * 2H20
	9.8 g	MgSO4 * 2H2O
		Fill up to 2000 mL with milliQ water
		Stir and autoclave (1 L suffices for 60 L 1x E3)
**E3 1x**		
	165 mL	60x E3
	10 L	milliQ water

A	B	C
**Pronase**		
Aqueous solution stable for 1 y at -20°C	5 g	Pronase (from Roche)
Shelf life: as indicated	167 mL	milliQ water
1. Mix 5 g Pronase with 167 mL with stirrer **under hood** until pronase is dissolved.<br>2. Store at -20°C in 1 mL aliqouts.		
**PTU**		N-Phenylthiourea (e.g. P7629-10 g)
Store powder in toxic material area (Shelf life: indefinite)	304 mg	PTU
Option1: **10x**		=10x stock → Fill up to 1000 mL with milliQ water
		Stir overnight in fumehood (toxic!), cover in aluminumfoil
Option2: **2500x** first dissolve in DMSO & store aliquots at 4°C	750 mg	PTU
	10 mL DMSO	Stir overnight, cover in aluminumfoil
		=2'500X stock → add 400 µL to 1 L E3 (0.003% PTU)
**Tricaine/Mesab**		
(= 4000 mg/L)	4g	Tricaine/Mesab
		Fill up with milliQ water to 1000 mL
		Adjust pH at 7.0
		Store at -20°C

Part 0: Material preparation

1 Media: prepare all required media according to Table SP1 (E3 zebrafish water, PTU to avoid pigmentation, pronase for dechorionation, tricaine for anesthetizing).

2 Hair loop manipulators (Fig. SP1A): tape (e.g. using 19 mm wide tape) a piece of hair as a 4-7 mm loop as an extension onto e.g. the wooden end of a cotton swab (ROTILAB, 150mm long) (see image of hairloop tool).

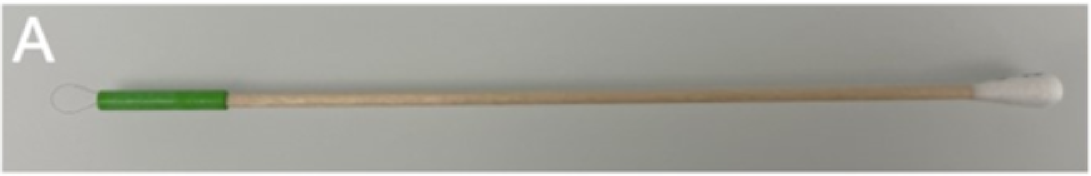

Hair loop manipulators (Fig. SP1A)

3 Needles (Fig. SP1B): Using the micropipette puller device, pull filamented borosilicate glass microcapillary injection needles (Science Products, 0.58mm diameter, GB100F-10) with the following settings: air pressure=500, heat=609, pull=200, velocity=100, time=50.

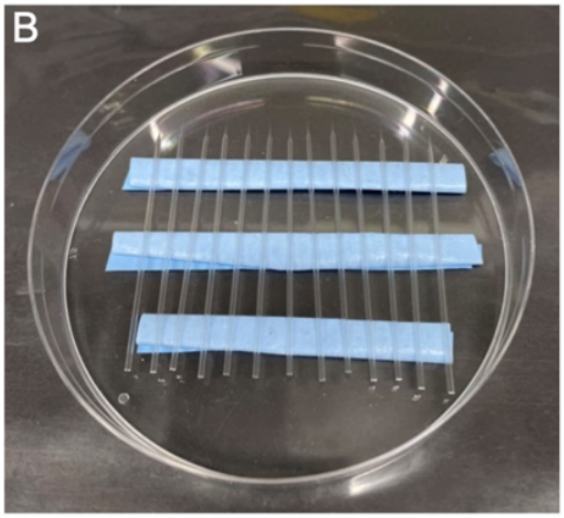

Pulled needles. (Fig. SP1B)

4 Agarose molds (Fig. SP1C right): prepare as many agarose molds as there are treatments using a negative rubber mold (Fig. SP1C left):

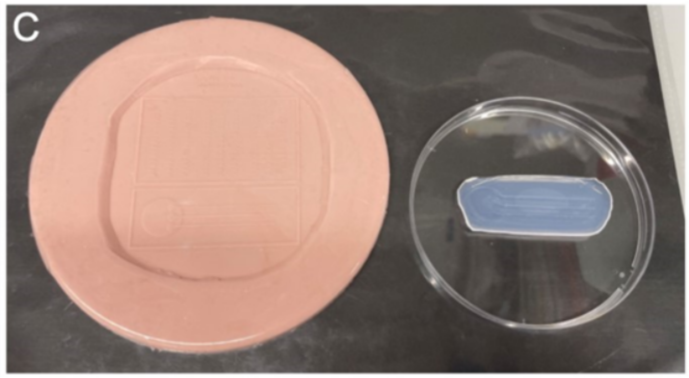

Agarose molds (Fig. SP1C right) a negative rubber mold (Fig. SP1C left)

4.1 Heat 1.5% agarose in 100 mL ddH2O in a beaker in a microwave in 00:00:30 bursts until the liquid is completely clear (no turbidity visible)

4.2 Carefully pour the heated agarose onto the rubber mold with channels from Ellett & Irimia [29] avoiding the formation of any bubbles.

4.3 Once it has solidified, peel the agarose mold off the rubber mold.

4.4 Store at 4 °C in a petri dish wrapped in parafilm until use.

Note

Heated agarose can be stored at 60 °C in between making several molds to prevent cooling/solidification.

Part 1: Zebrafish pre-experiment preparation (1 day post fertilization (dpf))

5 In the morning, move the zebrafish to a petri dish (LGG Labware, 90x16 mm, sterile) filled with about 20 mL E3 zebrafish water plus N-Phenylthiourea (PTU) (final PTU concentration: 0.003%) to avoid their pigmentation (see Table 1 on how to prepare E3 water and PTU).

6 In the evening, add 20 µL pronase (30 mg/mL) to the zebrafish (final pronase concentration: 0.03 mg/mL) and mix well to ensure that they will dechorionate (hatch from the chorion, which is the protective membrane around the zebrafish embryo) until the next morning.

Part 2: Preparation of bacterial suspensions (2 dpf)

7 Take fluorescently tagged bacterial species from 25% glycerol stocks that were stored at -80 °C and either streak them on an agar plate to pick a single colony (after overnight

growth at 37 °C) for the following inoculation or directly inoculate them into 10 mL fresh LB medium.

8 Grow Overnight at 37 °C and 170 rpm with aeration until they reach the stationary phase.

- Depending on the biological question being asked, exponentially growing cells could also be used.

9 The next day, wash cells by centrifuging them at 7500 rcf, 00:03:00 . Discard the supernatant and replace it with 0.8% NaCl to resuspend the cell pellet.

10 Repeat step 9.

11 Measure the optical density at a wavelength of 600 nm and adjust it to reach the desired cell numbers per bacterial species (to be determined in a pre-experiment).

12 Mix 9 µL per bacterial culture or 0.8% NaCl solution (as control) with 1 µL of 0.5% phenol red (final concentration: 0.05%) to visualize whether an infection is successful and remains local in the inner ear structure, the otic vesicle, of the zebrafish.

> **Note**
>
> For multispecies infections, mix different bacterial species (e.g. in a 1:1 ratio) directly before loading the needle (i.e. before step 3 of part 3) to minimize interactions between them before reaching the host. Then proceed by adding 1 µL of 0.5% phenol red to the 9 µL mixed bacterial culture.

Part 3: Zebrafish infections into the otic vesicle (2 dpf)

13 Add approximately 9 mL of E3 zebrafish water with PTU (final PTU concentration: 0.003%) and 10 drops of the thawed anesthetic tricaine (ethyl 3-aminobenzoate methanesulfonate salt analytical standard, 4000 mg/L) using a Pasteur pipette (LLG Labware, 3 mL, unsterile) into a small petri dish (Greiner, 60x15mm, sterile).

14 Add 10-20 2 dpf zebrafish into this dish to anesthetize them (Fig. SP2A).

> **Note**
>
> Careful: try to add as little liquid as possible when transferring the zebrafish into the small petri dish to avoid diluting the tricaine concentration.

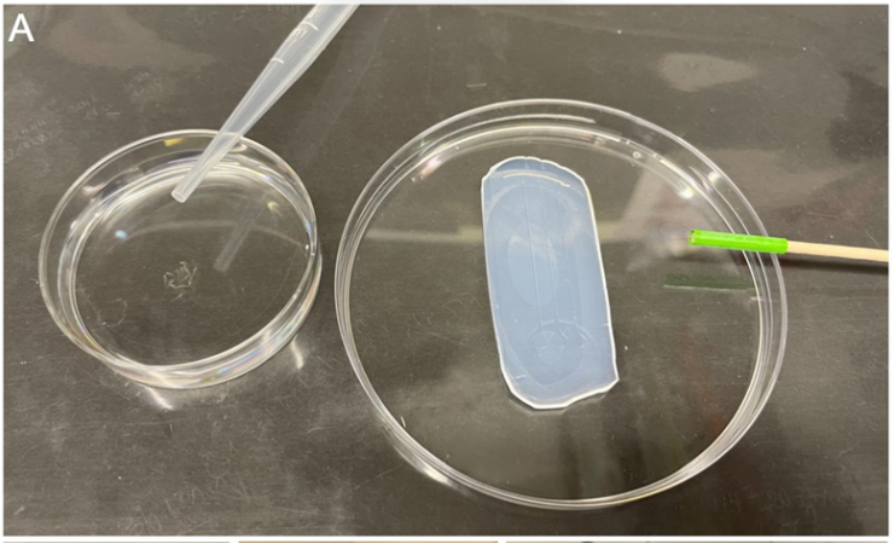

Figure SP2A. On the left are 2-days-postfertilization zebrafish in a small petri dish containing an anesthetizing solution (tricaine). A Pasteur pipette can be seen on top for moving the zebrafish from the petri dish onto the agarose mold. On the right is an agarose mold (placed on an inverted lid of a petri dish) and a hair loop manipulator.

15 For the first treatment, fill a glass needle (pulled borosilicate glass microcapillary injection needle (Science Products, 0.58mm diameter, GB100F-10)) with 10 µL of the 0.05% phenol red and 0.8% NaCl (control) or bacterial solution using a microloader tip (Eppendorf).

16 Put the filled glass needle into the micromanipulator, screwing it tight but not too tight to avoid breaking.

17 Position the needle in the field of view of the stereomicroscope using the micromanipulator.

18 Going from the lowest to the highest magnification, focus on the tip of the glass needle.

19 Once it is possible to distinguish the two walls of the needle at a final magnification of 56X, break off the tip of the glass needle using high-precision tweezers (e.g., RubisTech Switzerland, model 5-SA)(Fig. SP2C).

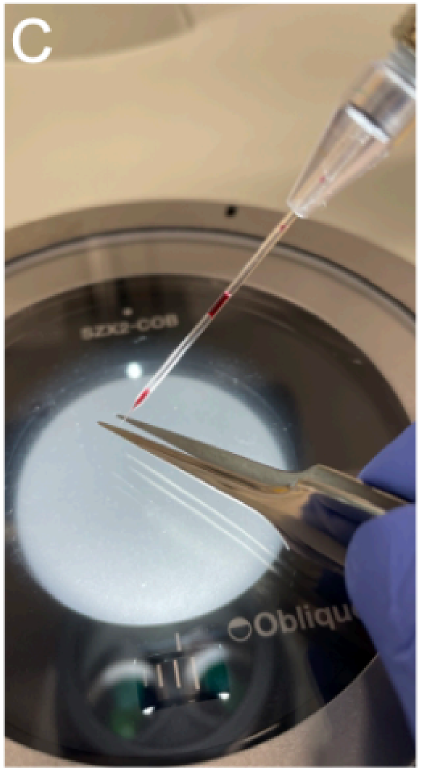

Figure SP2C. Breaking off the needle with fine tweezers using a stereomicroscope. The needle is held by a micromanipulator

20 Connect the injection pump with the micromanipulator (and thus the glass needle) via the tubing of the pump.

21 Put a drop of oil (VWR Chemicals, 10S) on a microscopy slide with a cavity (76x26 mm) that sits on an inverted lid of a petri dish (LGG Labware, 90x16 mm, sterile) and place it under the stereomicroscope.

22 Move the needle into the drop of oil using the micromanipulator and press ‘Clean’ on the injection pump to move the liquid into the tip of the needle.

23 Determine the desired drop size by injecting drops into the oil and adjusting either the injection time (to between 00:00:01 - 00:00:05) or injection pressure (to between 200-400 hPa). In case the solution in the needle gets aspirated back in or leaks, adjust the injection back pressure (to between 20-30 hPa). At a magnification of 56X, a drop that has the diameter of five stripes on the ruler corresponds to a volume of 1 nL. This can be determined because the liquid to inject will be a perfect sphere within the oil.

Note

Careful: the drop sinks to the bottom and will not be a perfect sphere anymore once it hits the bottom.

24 Whenever the needle is loaded and not in use, leave it in the oil to prevent evaporation of the liquid and the clogging of the needle.

25 Put a few drops of the E3-PTU-tricaine solution from the petri dish onto the agarose mold.

26 Make sure to wet the channels that will be used for the injection (lateral for the otic vesicle) using a hair loop manipulator.

27 Move the 2 dpf zebrafish from the small petri dish onto the large circle of the agarose mold for loading using a Pasteur pipette.

28 Load the zebrafish into the channels using a hair loop manipulator, turning all zebrafish onto the same side so that the same otic vesicle can be injected and imaged later on (left or right otic vesicle)(Fig. SP2B).

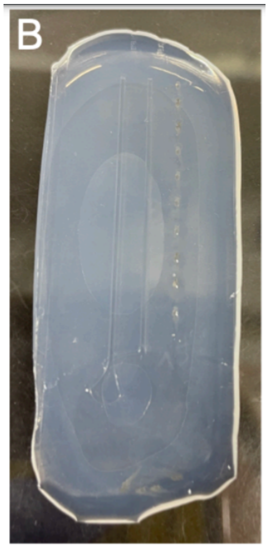

Figure SP2B. (B) An agarose mold with zebrafish aligned in the rightmost, the lateral channel.

29 Repeat step 14 to anesthetize the next batch of zebrafish for the same treatment. If it is a different treatment, this step is not necessary yet.

30 To determine the number of bacterial cells injected at the beginning of the injections, add a drop from the needle into 1 mL of 0.8% NaCl within a 1.5 mL Eppendorf tube.

31 Repeat step 30 to have at least duplicates for plating.

Note

Carefully move the glass needle into the NaCl solution of the Eppendorf tube without breaking it.

32 Place the mold under the objective of the stereomicroscope.

33 Using a magnification of 56X, position the needle into the otic vesicle and inject (Fig. SP2D).

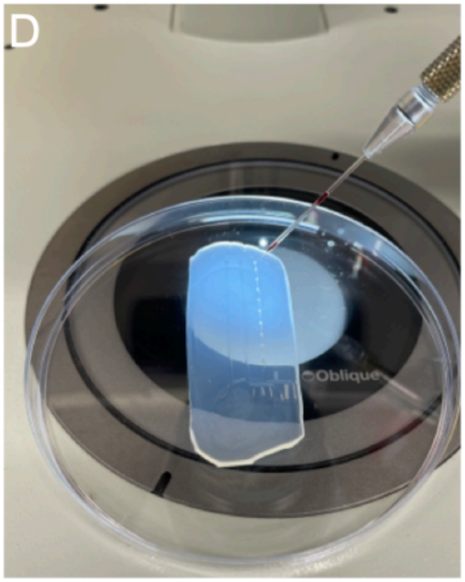

Figure SP2D. Injecting zebrafish using a stereomicroscope and a micromanipulator holding a glass needle.

Note

Careful: Make sure that the injection was applied correctly by checking whether the phenol red (bacterial) solution went into the otic vesicle and remained exclusively in there. Remove incorrectly injected zebrafish with the hair loop tool. Inject all zebrafish on the mold as described here.

34 Using a Pasteur pipette, aspirate the injected zebrafish and place them either individually into wells of a 48-well plate or five zebrafish individuals per well in a 24-well plate that is filled with E3 zebrafish water.

Note

Careful: first add some E3 zebrafish water onto the mold and then aspirate the zebrafish to prevent damaging them.

35 Repeat steps 27-34 if more zebrafish receiving the same treatment need to be injected.

36 To determine the number of bacterial cells injected at the end of the injections, add a drop from the needle into 1 mL of 0.8% NaCl within a 1.5 mL Eppendorf tube.

37 Repeat step 36 to have at least duplicates for plating.

Note

Carefully move the glass needle into the NaCl solution of the Eppendorf tube without breaking it.

38 Repeat steps 14-37 for each treatment.

39 Once all zebrafish from all treatments have been injected, check whether any zebrafish in the well-plate(s) have been damaged and remove them.

40 Incubate the well plate(s) at 28 °C either in the dark or with a 14:00:00 light and 10:00:00 dark cycle.

41 If necessary, further dilute and then plate 100 µL of the bacterial solution added to the 1 mL of 0.8% NaCl for all treatments in duplicates on 1.5% LB-agar plates to enumerate and confirm the infectious dose for each experiment.

Note

This step should also be used for pre-experiments to determine and define the number of cells to be injected per species.

42 Incubate plates at 37 °C (or the temperature required for your bacteria's growth) Overnight .

43 Count CFU from agar plates.
