# Supplementary material for: A new protocol for multispecies bacterial infections in zebrafish and their monitoring through automated image analysis
Source: PLoS One. 2024 Aug 8;19(8):e0304827. doi: 10.1371/journal.pone.0304827 (PMC11309447; doi:10.1371/journal.pone.0304827)
Supplement: S3 File — Also available on protocols.io. (PDF) [file pone.0304827.s003.pdf]

Protocol (B): Zebrafish embedding and imaging (3 dpf)

In 1 collection

RESERVED DOI:
10.17504/protocols.io.14egn6726l5d/v1

Désirée A. Schmitz[1,2], Tobias Wechsler[1], Hongwei Bran Li[1,3], Bjoern H. Menze[1], Rolf Kümmerli[1]

[1]Department of Quantitative Biomedicine, University of Zurich, Zurich, Switzerland;
[2]Department of Microbiology, Harvard Medical School, Boston, Massachusetts, USA;
[3]Athinoula A. Martinos Center for Biomedical Imaging, Massachusetts General Hospital, Harvard Medical School, Boston, Massachusetts, USA

Desiree Schmitz
Harvard Medical School

Protocol Info: Désirée A. Schmitz, Tobias Wechsler, Hongwei Bran Li, Bjoern H. Menze, Rolf Kümmerli . Protocol (B): Zebrafish embedding and imaging (3 dpf). **protocols.io** **https://protocols.io/view/protocol-b-zebrafish-embedding-and-imaging-3-dpf-dc472yzn**

Created: April 16, 2024

Last Modified: May 01, 2024

Protocol Integer ID: 99199

Abstract

This protocol details the zebrafish embedding and imaging.

Guidelines

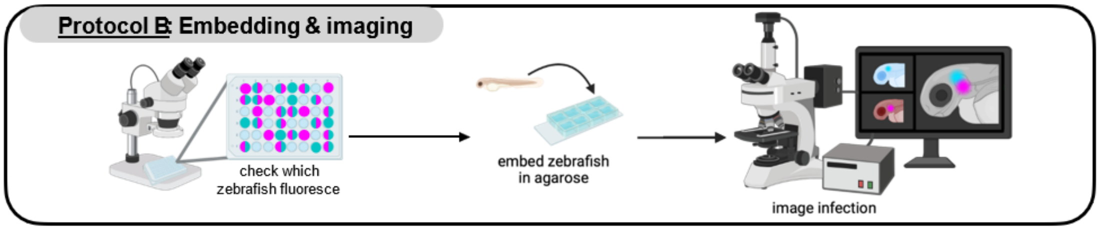

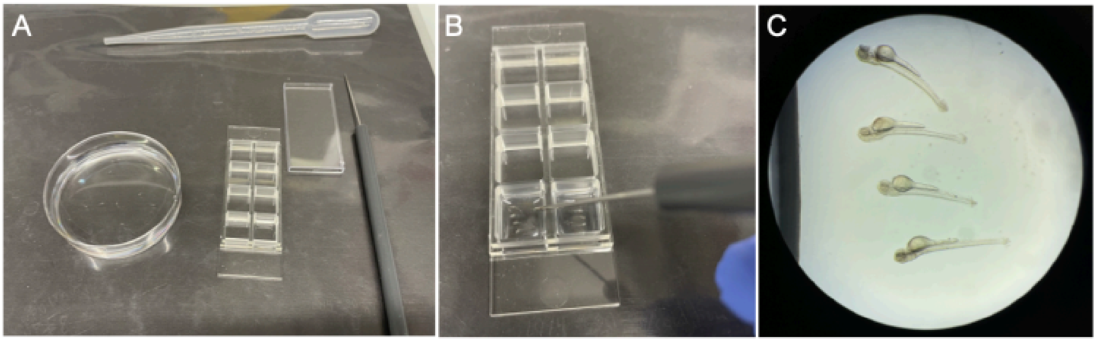

Figure SP3. Embedding zebrafish. (A) On the left are 3-days-post-fertilization (24 h post-infection) zebrafish in a small petri dish containing an anesthetizing solution (tricaine). Next to it is an Ibidi slide with the lid and a dissecting needle on its right side. On the top is a Pasteur pipette for moving zebrafish from the petri dish to the Ibidi µ slide. (B) Aligning zebrafish with a dissection needle in low-melting point agarose so that the infected otic vesicle is on the bottom. (C) View from a stereomicroscope to check whether zebrafish are properly aligned, facing the bottom with the otic vesicle thas has been infected (here: left otic vesicle).

Materials

Equipment:

For this protocol, you will need the following equipment:

- A stereomicroscope to embed zebrafish for imaging
- A heating block compatible with 1.5 mL Eppendorf tubes
- A widefield microscope with fluorescence filters that match the corresponding fluorophores of the tested bacterial species/ strains (e.g. Leica Thunder DMi8 widefield microscope with the Leica monochrome fluorescence DFC9000 GTC camera system)

Part 0: Material preparation

1 Prepare 1.5% low-melting point agarose by heating e.g. 1.5 g low-melting point agarose (Sigma, serial no: A9414) in 100 mL distilled water in a flask (minimal volume 150 mL) in 00:00:30 bursts in the microwave.

2 Once the solution is clear, i.e. no flocs or powder are visible anymore, aliquot into 1.5 mL Eppendorf tubes.

3 The day before embedding, prepare an Overnight culture of all bacteria used for injections. These will serve as a positive control.

Part 1: Embedding zebrafish in low-melting point agarose

4 Heat 1.5% low-melting point agarose in 1.5 mL Eppendorf tube aliquots to 90 °C in a heating block.

5 Once the agarose has melted, reduce the heat to 42 °C .

6 Check fluorescence in the otic vesicle of individual zebrafish using any microscope with sufficient fluorescence sensitivity and magnification. This step is only needed if the goal is to solely image zebrafish with an ongoing infection. These fishes can then be picked selectively based on their fluorescent signal in the otic vesicle.

7 Prepare a sufficiently large volume of anesthetic solution to use for all treatments: For this, add approximately 9 mL of E3 zebrafish water with PTU (final PTU concentration: 0.003%) and 10 drops of the thawed anesthetic tricaine (ethyl 3aminobenzoate methanesulfonate salt analytical standard, 4000 mg/L) using a Pasteur pipette (LLG Labware, 3 mL, unsterile) into a small petri dish (Greiner, 60x15mm, sterile).

8 For each treatment, move a few drops of the solution from the previous step into a separate small petri dish. This allows anesthetizing each treatment group individually to avoid cross-contamination.

9 Move the zebrafish of the first treatment into the anesthetic liquid in a designated petri dish (Fig. SP3A).

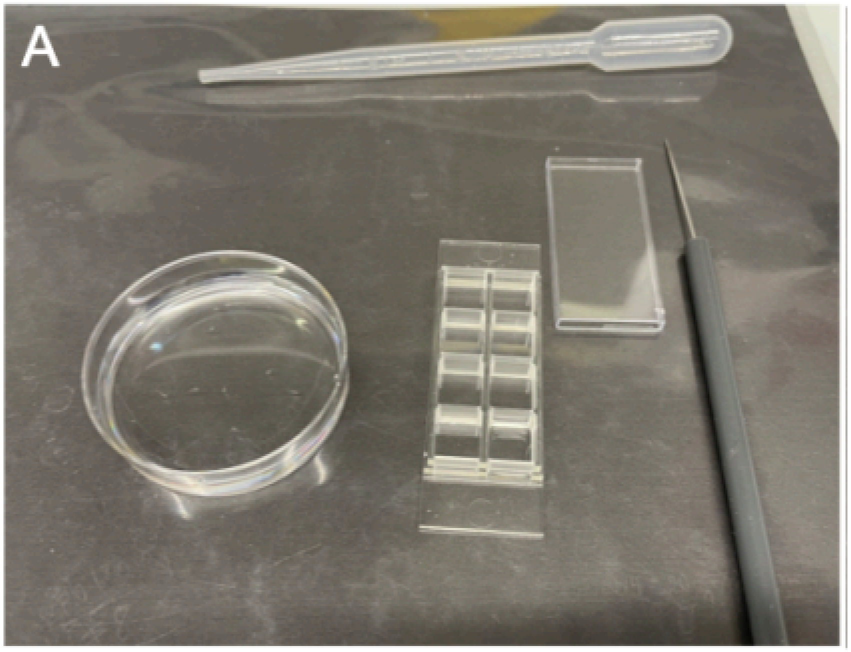

Figure SP3A. On the left are 3-days-post-fertilization (24 h postinfection) zebrafish in a small petri dish containing an anesthetizing solution (tricaine). Next to it is an Ibidi µ slide with the lid and a dissecting needle on its right side. On the top is a Pasteur pipette for moving zebrafish from the petri dish to the Ibidi µ slide.

Note

Careful: try to add as little E3 zebrafish water as possible with the zebrafish into the petri dishes to avoid diluting the concentration of the anesthetic.

10 Add 4-5 zebrafish of one treatment into an 8-well Ibidi µ slide (Vitaris, serial no: 80827-IBI) (Fig. SP3B).

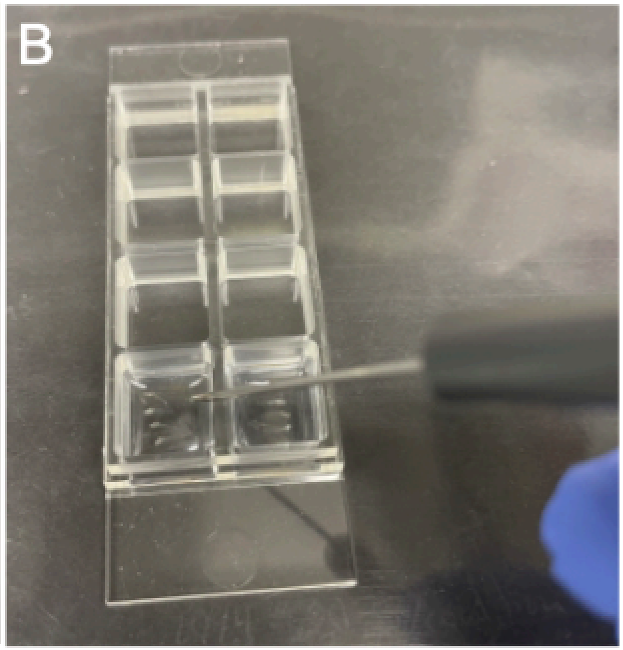

Figure SP3B. Aligning zebrafish with a dissection needle in low-melting point agarose so that the infected otic vesicle is on the bottom.

Note

Careful: again, try to add as little liquid as possible with the zebrafish into the well to avoid diluting the agar concentration.

11 Using a Pasteur pipette, add about 5 drops of the low-melting point agarose (from the 42 °C in the heating block) to the zebrafish in a well.

Note

The more agarose is added, the more time is usually required for positioning the zebrafish. However, the less agarose is added, the faster it will solidify.

12 Using a dissecting needle, mix the agarose and the small amount of liquid that was transferred with the zebrafish in the well so that the agarose concentration is the same throughout.

Note

Careful: do not damage the zebrafish with the needle.

13 Position the zebrafish with the dissecting needle so that the injected otic vesicle (see protocol A) is on the bottom of the slide to enable the use of an inverted widefield microscope for imaging (Fig. SP3C). Do this step with the naked eye as well as checking under a stereomicroscope.

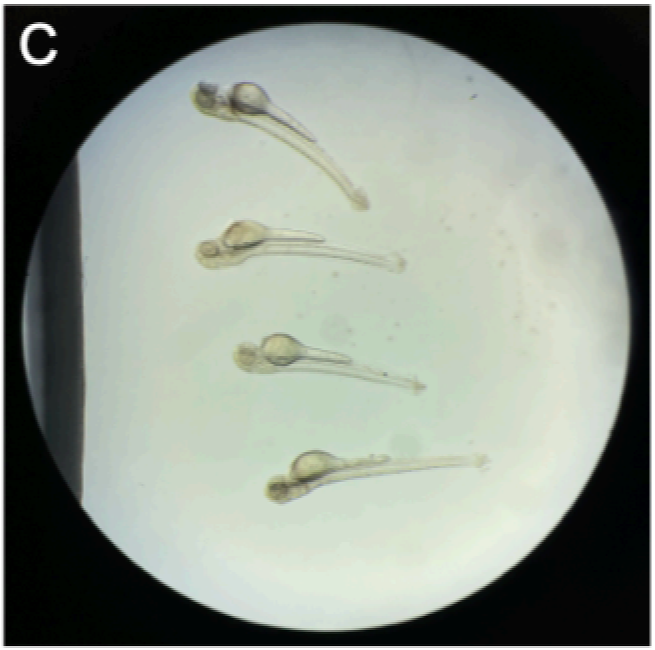

Figure SP3C. View from a stereomicroscope to check whether zebrafish are properly aligned, facing the bottom with the otic vesicle thas has been infected (here: left otic vesicle).

Note

Press the zebrafish onto the bottom of the well to reduce the working distance for the microscope, i.e., to improve the quality of the images.

14 Wait approximately 00:05:00 until the agarose has completely hardened.

15 Add E3 zebrafish water very carefully on top to ensure a constant supply of moisture.

16 Repeat steps 9-15 for all zebrafish that need to be embedded for imaging.

17 Add a positive control for each tagged bacterial species/strain, i.e., ~ 10 µL Overnight culture mixed with agarose into one well per species.

Part 2: Imaging the inner ear structure (otic vesicle) of zebrafish with a widefield microscope

18 Place up to 4 Ibidi slides into the slide holder in a widefield microscope.

19 Set up the brightfield and all required fluorescence channels, e.g., GFP (excitation at 475 nm & emission 520 nm), and mCherry (excitation at 555 nm & emission at 605 nm).

20 Set the positions of all otic vesicles with a 20X objective that has a long working distance (minimum 1 mm).

21 Image all saved positions of zebrafish otic vesicles from the previous step within the brightfield and fluorescence channels.

22 Save the images as .tiff files for further processing, also keeping the original images from the imaging software.
