# Supplementary material for: A new protocol for multispecies bacterial infections in zebrafish and their monitoring through automated image analysis
Source: PLoS One. 2024 Aug 8;19(8):e0304827. doi: 10.1371/journal.pone.0304827 (PMC11309447; doi:10.1371/journal.pone.0304827)
Supplement: S4 File — Also available on protocols.io. (PDF) [file pone.0304827.s004.pdf]

Protocol (C): Automated segmentation of the otic vesicle and image analysis

In 1 collection

RESERVED DOI:
10.17504/protocols.io.bp2l6219dgqe/v1

Désirée A. Schmitz[1], Tobias Wechsler[1], Hongwei Bran Li[1,2], Bjoern Menze[1], Rolf Kümmerli[1]

[1]Department of Quantitative Biomedicine, University of Zurich, Zurich, Switzerland;
[2]Massachusetts General Hospital & Harvard Medical School, Harvard University, Boston, USA

Desiree Schmitz
Harvard Medical School

Protocol Info: Désirée A. Schmitz, Tobias Wechsler, Hongwei Bran Li, Bjoern Menze, Rolf Kümmerli . Protocol (C): Automated segmentation of the otic vesicle and image analysis. **protocols.io** **https://protocols.io/view/protocol-c-automated-segmentation-of-the-otic-vesi-dc482yzw**

Created: April 16, 2024

Last Modified: May 01, 2024

Protocol Integer ID: 99200

Abstract

This protocol details the automated segmentation of the otic vesicle and image analysis.

Materials

For this protocol, you will need the following scripts and software:

- Docker and the docker image (docker pull branhongweili/dqbm_cell_seg:v3.1) to run the pre-trained segmentation model.
- Depending on the operating system, either use the segmentation.sh shell script (for MacOS/Ubuntu) or the segmentation.ps1 PowerShell script (for Windows).
- An installation of FIJI.
- The check_measure.py script.
- An installation of R.
- The co_localization.R script.

Automated segmentation of the otic vesicle and image analysis

1 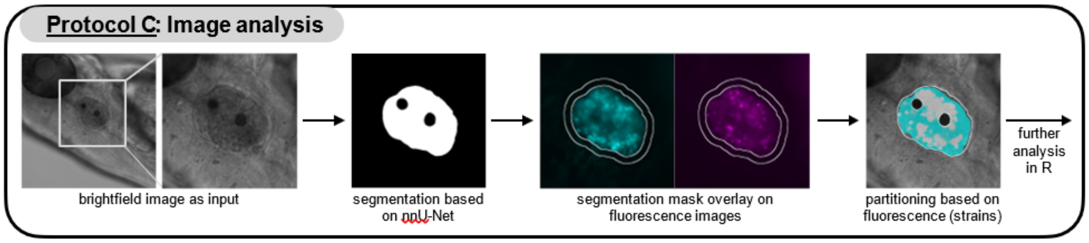

2 Organize your images in a folder with two subfolders:

- one for the brightfield images named 'Images',
- one for all fluorescence images named 'Fluor'.

The image file names have to contain the slice number with a Z as a prefix and the channel number (0-based) with a C as a prefix (e.g., PositionName_Z00_C00.tif). For example, for measuring two different fluorophores and the brightfield image, we have three channels, C00 (e.g., brightfield), C01 (e.g., GFP), C02 (e.g., mCherry). We include an example script to convert Leica image files accordingly (sort_images_lif.py), which can be used as an example of how to organize all images automatically as described.

3 For the segmentation of the otic vesicle, run the segmentation.sh (segmentation.ps1 for Windows) script with the previously created directory as input (e.g. for Mac: in Terminal). Make sure the shell script is executable (e.g. for Mac: by typing

```
chmod +x segmentation.sh
```

in Terminal). The script will start a docker container and the segmentation model on the specified brightfield images.

4 In the same directory that contains your brightfield (Images) and fluorescence (Fluor) images, a directory named output_real_value is created that contains the segmentation masks.

5 Start FIJI and open the check_measure.py script.

6 Run the script and select the directory with your brightfield (Images) and fluorescence (Fluor) images and segmentation masks (output_real_value) as input.

7 FIJI will display the brightfield images and the corresponding segmentation. In the ROI Manager window untick the box "Show All" and select one of the ROIs in the list to only show the segmentation for a single ROI. Go through the ROI sequence and delete inaccurate segmentations.

8 Complete the manual review of the segmentations by pressing OK.

9 The script creates six files:

9.1 an image with an overlay of the strain partitioning based on fluorescence (e.g. Sample1_overlay.tif),

9.2 a text file containing the log to check what has been done up until here (e.g. Sample1_log.txt)

9.3 a zip file with the ROIs (Sample1_rois.zip)

9.4 a table with fluorescence values within the whole otic vesicle (mean and integrated density for both GFP and mCherry; e.g. Sample1 ov_data.csv)

9.5 A table with the size of the area occupied by tagged strains (e.g. Sample1_strain_count.csv) → e.g. needed to calculate co-localization

9.6 A table with the fluorescence values of individual pixels within the otic vesicle (e.g. Sample1 pixel_data.csv).

Note

Since the zebrafish shows inherent auto-fluorescence, we introduced a threshold value to delineate bacterial occupation from auto-fluorescence. The threshold value for bacterial occupation is defined as twice the fluorescence value observed in the surrounding tissue of the otic vesicle (a layer of approximately 16 µm, corresponding to 50 pixels).

10 Use the accompanying R-script to visualize the size of the area occupied by each tagged bacterial species and the overlap between the two species to quantify their co-localization (script: co_localization.R).

11 Check whether the fluorescence images in FIJI correspond to the values in R by dropping the Fluor folder into FIJI and comparing it to the R plot.
