# Supplementary figures and images for: A new protocol for multispecies bacterial infections in zebrafish and their monitoring through automated image analysis
Source: PLoS One. 2024 Aug 8;19(8):e0304827. doi: 10.1371/journal.pone.0304827 (PMC11309447; doi:10.1371/journal.pone.0304827)

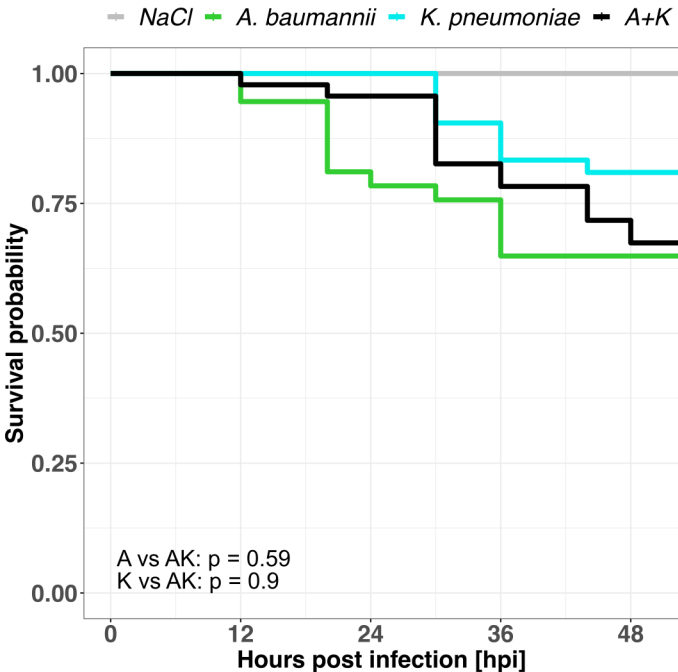

Supplement: S1 Fig — Survival of zebrafish with co-infections (black curve) and the corresponding mono-infections (colored curves). The mean number of CFU is 6850 for K. pneumoniae and 8600 for A. baumannii. Survival (y-axis) was monitored every 4–8 h for 54 h in total (x-axis). Control larvae were injected with a 0.8% NaCl solution. We conducted multiple pairwise comparisons using the log-rank test (alpha = 0.05, Benjamini-Hochberg p-value adjustment). Data are from two independent experiments with the following numbers of individual zebrafish per treatment: control, N = 48; A. baumannii mono, N = 37; K. pneumoniae mono, N = 42; mix, N = 46. (PDF) [file pone.0304827.s007.pdf]

*A. baumannii* + *K. pneumoniae*

**A**

1

5

10

Original  
images

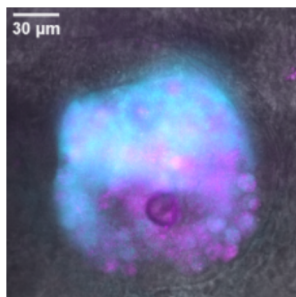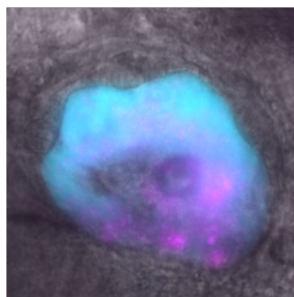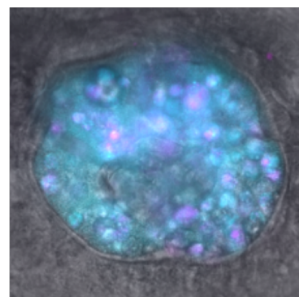

**B**

Segmented  
images

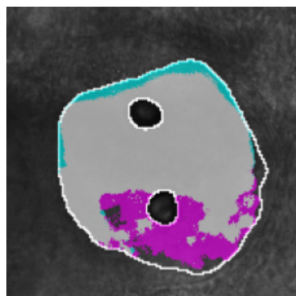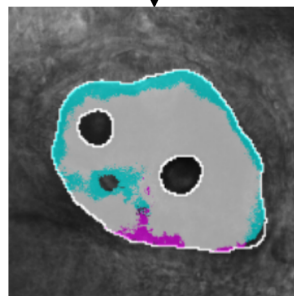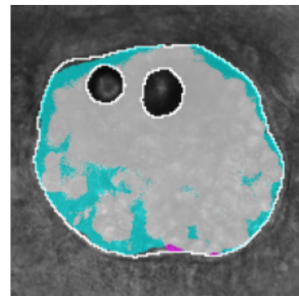

**C**

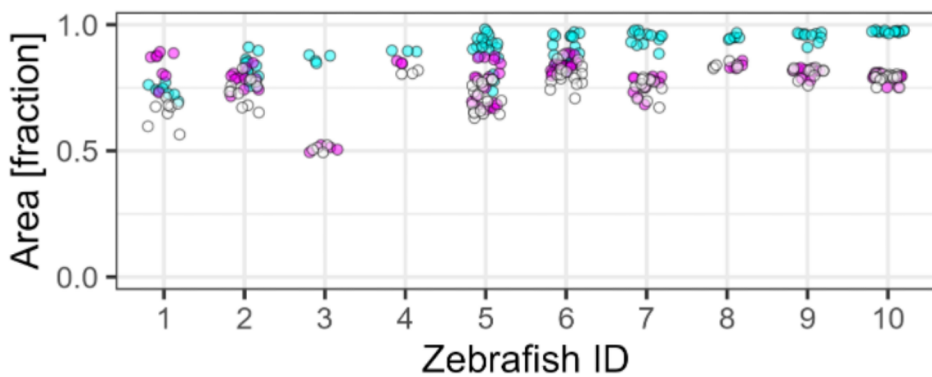

Supplement: S2 Fig — (A) Representative images of three individual zebrafish are shown as overlays of the brightfield, GFP (excitation at 475 nm & emission at 520 nm; shown in cyan), and mCherry (excitation at 555 nm & emission at 605 nm; shown in magenta) image. (B) Masks obtained from automated segmentation are shown in white. The partitioning of the bacterial strains is done using the fluorescence signal. Since the zebrafish show some inherent auto-fluorescence, bacterial occupation is defined by a fluorescence value that is twice as high as the fluorescence value observed in the surrounding tissue of the otic vesicle (a layer of approximately 16 μm, corresponding to 50 pixels). (C) Quantitative image analysis showing the relative area of the otic vesicle that is occupied by the co-infecting pathogens (y-axis) across zebrafish individuals (x-axis), ordered from lowest to highest bacterial occupation. Magenta and cyan fractions respectively represent the area occupied by either K. pneumoniae or A. baumannii, with the mean number of CFU injected being 8100 and 7400, respectively. The grey fraction (open circles) represents the area simultaneously occupied by both pathogens. Each data point represents a z-slice imaged from the respective zebrafish ID. The data shown are from one experiment with n = 10. (PDF) [file pone.0304827.s008.pdf]
