# Supplementary material for: A new protocol for multispecies bacterial infections in zebrafish and their monitoring through automated image analysis
Source: PLoS One. 2024 Aug 8;19(8):e0304827. doi: 10.1371/journal.pone.0304827 (PMC11309447; doi:10.1371/journal.pone.0304827)
Supplement: S3 Fig — (A) Representative images of individual zebrafish per pathogen are shown as overlays of the brightfield, GFP (excitation at 475 nm & emission at 520 nm; shown in cyan), and mCherry (excitation at 555 nm & emission at 605 nm; shown in magenta) image. (B) Masks obtained from automated segmentation are shown in white. Since the zebrafish show some inherent auto-fluorescence, bacterial occupation is defined by a fluorescence value that is twice as high as the fluorescence value observed in the surrounding tissue of the otic vesicle (a layer of approximately 16 μm, corresponding to 50 pixels). (C) Quantitative image analysis showing the relative area of the otic vesicle that is occupied by a pathogen (y-axis) across zebrafish individuals (x-axis), ordered from lowest to highest bacterial occupation. The GFP signal is shown in cyan (A. baumannii and K. pneumoniae on left) and the mCherry signal in magenta (P. aeruginosa and K. pneumoniae on right). The mean number of CFU injected from left to right is as follows: 7400, 9200, 13200, and 8100. The grey fraction (open circles) represents the area when both signals overlap. Each data point represents a z-slice imaged from the respective zebrafish ID. The data shown are from two individual experiments, with n = 5 for each mono-infection. (PDF) [file pone.0304827.s009.pdf]

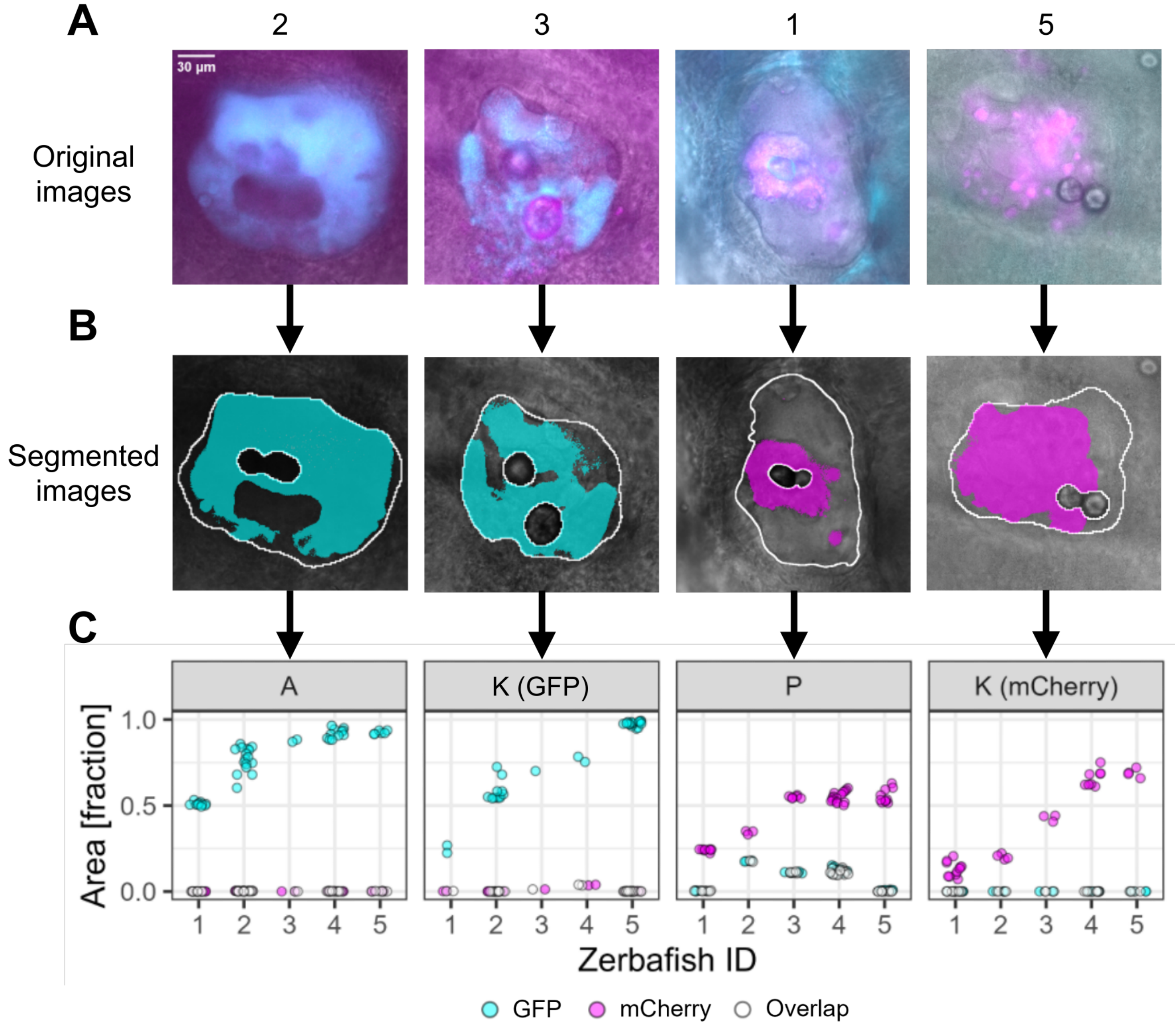
A
2
3
1
5
30 µm
Original images
B
Segmented images
C
A
K (GFP)
P
K (mCherry)
Area [fraction]
1.0
0.5
0.0
1
2
3
4
5
Zerbafish ID
GFP
mCherry
Overlap
