# Supplementary material for: A new protocol for multispecies bacterial infections in zebrafish and their monitoring through automated image analysis
Source: PLoS One. 2024 Aug 8;19(8):e0304827. doi: 10.1371/journal.pone.0304827 (PMC11309447; doi:10.1371/journal.pone.0304827)
Supplement: Attachment — Submitted filename: revision.pdf [file pone.0304827.s010.pdf]

In this study, the Authors described the protocol they develop for establishing and tracking single and multispecies bacterial infections in the otic vesicle of the zebrafish by imaging. They also generate an image analysis pipeline for the automated segmentation of this structure, and quantify pathogen load through fluorescence intensity.

Essentially, this study is based on the description of a new methodology developed in a zebrafish model of bacterial co-infection. In my opinion, the proposed methodology is not as innovative or faster or more easily usable compared to existing techniques. Therefore, the proposed work, although the imaging analysis technology described is novel, does not present sufficiently innovative details is more suitable for a methodological journal. Furthermore, from an experimental standpoint, there would be many points to implement as major revisions listed below:

1) For infections fresh bacterial cultures (in exponential phase) might be used as they in this phase they are "healthier" and more virulent. Pre-inocula grown overnight (stationary phase) contain many cellular debris, membranes, and LPS released by dead bacteria that could be confounding.

2) The Authors use widefield microscopy instead of confocal (i.e., they do not have a 3D image but only XY). So, why include the infected embryos to be photographed in LM agarosio? This is time consuming and is similar to the standard imaging techniques. Sometimes it is also sufficient to photograph them in E3+tricaine. In other words, if they want to pass it off as a quick and less tricky protocol, this step complicates things a lot.

3) In the section "Time for infections, imaging, and analysis highly depends on the number of treatments and zebrafish injected," it seems a bit approximate, redundant, and inconsistent. Rather than having a dedicated paragraph, if the Authors really want to indicate the timing of the protocols, I would include them in the text as they explain them.

4) The Authors incubate the embryos with a 14-hour light and 10-hour dark cycle?? Please clarify this point as usually embryos are kept in dark in the incubator.

5) Survival experiments (from which they derive Kaplan-Meier graphs) are only done in biological duplicate, not triplicate.

6) There is no correspondence between the fluorescence values the Authors obtain from the analysis using their script on ImageJ and the survival data (during co-infections). For example, *P. aeruginosa* is always more lethal in terms of survival but consistently shows lower fluorescence compared to *Klebsiella* and *Acinetobacter baumanii*. How do they explain this?

7) The amount of CFU injected for the different bacterial strains are higher compared to those used in similar model of infection in zebrafish. A dose dependent assay to justify the doses is necessary or a discuss possible motivation (i.e. the injection in the otic vescisle requires higher bacterial load to induce inflammation in comparison to other districts commonly used in zebrafish embryo/larvae?).

8) In the limitations (last paragraph), the Authors mentioned they cannot set the CFU/embryos injected properly. There is a great variability from experiment to experiment. Even though they mention discarding experiments with too much CFU/embryo variability, this remains a significant limitation because if the initial inoculum is incorrect, it can alter the development of bacterial co-infection (the ratio between the two species). Several works properly described different ways to calculate precisely the injected CFU.

9) I understand the rationale is to study the interaction in terms of local infective capacity (in an organ) between two bacteria belonging to different species or genera during a co-infection. However, the only output the Authors extract from the protocol is the fluorescence value of each bacterium and the overlapped signal. They do not experimentally explain or at least discuss how they connect this single output (along with embryo survival) to a greater fitness of the bacterium in a co-infection. The model is made more robust and appealing by the fact that there is interaction even with the innate immunity in that district.

10) The introduction misses the mark. It fails to describe the zebrafish as a model for bacterial infection, instead delving into the broader history of zebrafish as a model organism. It's irrelevant to discuss the transparency of zebrafish embryos or their non-animal status until five days post-fertilization.
