# Supplementary material for: A new protocol for multispecies bacterial infections in zebrafish and their monitoring through automated image analysis
Source: PLoS One. 2024 Aug 8;19(8):e0304827. doi: 10.1371/journal.pone.0304827 (PMC11309447; doi:10.1371/journal.pone.0304827)
Supplement: Attachment — Submitted filename: ResponseToReviewers_PONE_Schmitz_etal.docx [file pone.0304827.s011.docx]

**Response to Editor & Reviewers**

Dear Academic Editor Michael Schubert,

Dear Reviewers,

On behalf of all the co-authors, we would like to thank you for taking the time to review our manuscript and for making valuable suggestions for improvement. Please find all comments and requirements addressed in blue text.

**Academic Editor/Journal requirements**

1. Please ensure that your manuscript meets PLOS ONE's style requirements, including those for file naming. The PLOS ONE style templates can be found at

<https://journals.plos.org/plosone/s/file?id=wjVg/PLOSOne_formatting_sample_main_body.pdf> and

<https://journals.plos.org/plosone/s/file?id=ba62/PLOSOne_formatting_sample_title_authors_affiliations.pdf>

We followed the given style requirements.

2. Thank you for stating the following financial disclosure:

“Swiss National Science Foundation (grant no. 31003A_182499 and 310030_212266 to RK).”

Please state what role the funders took in the study.  If the funders had no role, please state: "The funders had no role in study design, data collection and analysis, decision to publish, or preparation of the manuscript."

If this statement is not correct you must amend it as needed.

Please include this amended Role of Funder statement in your cover letter; we will change the online submission form on your behalf.

The Role of Funder statement is included in the cover letter as requested.

3. Please note that your Data Availability Statement is currently missing the DOI/accession number of each dataset OR a direct link to access each database. If your manuscript is accepted for publication, you will be asked to provide these details on a very short timeline. We therefore suggest that you provide this information now, though we will not hold up the peer review process if you are unable.

We have added the links to access both the

- survival data (<https://doi.org/10.6084/m9.figshare.25669311>) and

- imaging data (<https://www.ebi.ac.uk/biostudies/bioimages/studies/S-BIAD1159?key=ff1fb1fc-54f1-47fc-b14d-c1bf4c41d33c>) in the manuscript.

4. We note that Figure 1 in your submission contain copyrighted images. All PLOS content is published under the Creative Commons Attribution License (CC BY 4.0), which means that the manuscript, images, and Supporting Information files will be freely available online, and any third party is permitted to access, download, copy, distribute, and use these materials in any way, even commercially, with proper attribution. For more information, see our copyright guidelines: <http://journals.plos.org/plosone/s/licenses-and-copyright>.

We require you to either (1) present written permission from the copyright holder to publish these figures specifically under the CC BY 4.0 license, or (2) remove the figures from your submission:

1. You may seek permission from the original copyright holder of Figure 1 to publish the content specifically under the CC BY 4.0 license.

We recommend that you contact the original copyright holder with the Content Permission Form (<http://journals.plos.org/plosone/s/file?id=7c09/content-permission-form.pdf>) and the following text:

“I request permission for the open-access journal PLOS ONE to publish XXX under the Creative Commons Attribution License (CCAL) CC BY 4.0 (<http://creativecommons.org/licenses/by/4.0/>). Please be aware that this license allows unrestricted use and distribution, even commercially, by third parties. Please reply and provide explicit written permission to publish XXX under a CC BY license and complete the attached form.”

Please upload the completed Content Permission Form or other proof of granted permissions as an "Other" file with your submission.

In the figure caption of the copyrighted figure, please include the following text: “Reprinted from [ref] under a CC BY license, with permission from [name of publisher], original copyright [original copyright year].”

2. If you are unable to obtain permission from the original copyright holder to publish these figures under the CC BY 4.0 license or if the copyright holder’s requirements are incompatible with the CC BY 4.0 license, please either i) remove the figure or ii) supply a replacement figure that complies with the CC BY 4.0 license. Please check copyright information on all replacement figures and update the figure caption with source information. If applicable, please specify in the figure caption text when a figure is similar but not identical to the original image and is therefore for illustrative purposes only.

We have included the publication license from BioRender for Figure 1 and Gena Louque, your Peer Review Operations Specialist, has confirmed with BioRender that their images can be published under your CC BY 4.0 license.

5. We note you have not yet provided a [protocols.io](http://protocols.io) PDF version of your protocol and/or a [protocols.io](http://protocols.io) DOI. When you submit your revision, please provide a PDF version of your protocol as generated by [protocols.io](http://protocols.io) (the file will have the [protocols.io](http://protocols.io) logo in the upper right corner of the first page) as a Supporting Information file. The filename should be S1_file.pdf, and you should enter “S1 File” into the Description field. Any additional protocols should be numbered S2, S3, and so on. Please also follow the instructions for Supporting Information captions [<https://journals.plos.org/plosone/s/supporting-information#loc-captions>]. The title in the caption should read: “Step-by-step protocol, also available on [protocols.io](http://protocols.io).”

Please assign your protocol a [protocols.io](http://protocols.io) DOI, if you have not already done so, and include the following line in the Materials and Methods section of your manuscript: “The protocol described in this peer-reviewed article is published on [protocols.io](http://protocols.io)([https://dx.doi.org/10.17504/protocols.io.[](https://dx.doi.org/10.17504/protocols.io.%5B)...]) and is included for printing purposes as S1 File.” You should also supply the DOI in the [Protocols.io](http://Protocols.io) DOI field of the submission form when you submit your revision.

If you have not yet uploaded your protocol to [protocols.io](http://protocols.io), you are invited to use the platform’s protocol entry service [<https://www.protocols.io/we-enter-protocols>] for doing so, at no charge. Through this service, the team at [protocols.io](http://protocols.io) will enter your protocol for you and format it in a way that takes advantage of the platform’s features. When submitting your protocol to the protocol entry service please include the customer code PLOS2022 in the Note field and indicate that your protocol is associated with a PLOS ONE Lab Protocol Submission. You should also include the title and manuscript number of your PLOS ONE submission.

We have uploaded our protocols on protocols.io, uploaded the PDF versions on the PLOS ONE submission form, and added the DOIs in the online submission form as well as in the manuscript.

Private link: <https://www.protocols.io/private/3E54EF54095611EFA6A10A58A9FEAC02>
DOI: dx.doi.org/10.17504/protocols.io.rm7vzjybxlx1/v1 (reserved DOI, publicly accessible upon publishing)

6. Please include captions for your Supporting Information files at the end of your manuscript, and update any in-text citations to match accordingly. Please see our Supporting Information guidelines for more information: <http://journals.plos.org/plosone/s/supporting-information>.

We have included all captions for the Supporting Information at the end of our manuscript.

**Reviewer #1**

In this study, the Authors described the protocol they develop for establishing and tracking single and multispecies bacterial infections in the otic vesicle of the zebrafish by imaging. They also generate an image analysis pipeline for the automated segmentation of this structure, and quantify pathogen load through fluorescence intensity.

Essentially, this study is based on the description of a new methodology developed in a zebrafish model of bacterial co-infection. In my opinion, the proposed methodology is not as innovative or faster or more easily usable compared to existing techniques. Therefore, the proposed work, although the imaging analysis technology described is novel, does not present sufficiently innovative details is more suitable for a methodological journal.

Thank you for your feedback regarding our study. We appreciate that you raise concerns, which have helped us to improve our manuscript. As your main concern is that our work is more suitable for a methodological journal, we would like to point out that “PLOS ONE *Lab Protocols*” is specifically made for this type of work. As PLOS ONE states on its website, their “Lab Protocols” articles *“offer authors the opportunity to share their peer-reviewed step-by-step protocols”.* This means an original research article combined with detailed protocols is aimed at “*improving the reproducibility and transparency of scientific research*”. Please find more information about PLOS ONE Lab Protocols [here](https://collections.plos.org/collection/lab-protocols/).

Please find below our detailed responses to each of your comments. Whenever we state line numbers, they refer to the clean manuscript version (i.e., not the tracked changes version).

List of required revisions:

1) For infections fresh bacterial cultures (in exponential phase) might be used as they in this phase they are "healthier" and more virulent. Pre-inocula grown overnight (stationary phase) contain many cellular debris, membranes, and LPS released by dead bacteria that could be confounding.

1) Thank you for this comment. We agree that cells harvested in the exponential phase might be “healthier”. However, infections in natural settings are probably rarely caused by exponentially growing cells. That is why we believe that both culturing options are valid, and we leave it to the experimenter to choose. **We have now added this discussion point to our protocol** (see protocol A part 2 step 8). Important to note is that we washed our bacterial cultures twice with a 0.8% NaCl solution to avoid any confounding effects of cellular debris, membranes, or LPS released by dead bacteria. Please see the detailed description in our protocol A part 2 steps 9-10.

2) The Authors use widefield microscopy instead of confocal (i.e., they do not have a 3D image but only XY). So, why include the infected embryos to be photographed in LM agarosio? This is time consuming and is similar to the standard imaging techniques. Sometimes it is also sufficient to photograph them in E3+tricaine. In other words, if they want to pass it off as a quick and less tricky protocol, this step complicates things a lot.

2) We used widefield microscopy for three reasons. First, it is faster than confocal microscopy, especially in the context of automated consecutive imaging of multiple fish samples. Second, high-content 3D imaging is very well possible. Note that we used z-stack imaging for the data shown in Fig. 4. Third, a widefield microscope is more widely available to researchers than a confocal microscope. In general, confocal microscopy takes longer for imaging than widefield microscopy and aims at a well-resolved 3D image. In contrast, the goal of our protocol is a more high-throughput approach, which is why we used a widefield microscope. While you are correct that embedding zebrafish in LM agarose is more time-consuming than putting them in E3+tricaine, the imaging itself is faster as soon as you have many samples. This is due to no waiting time for imaging between samples. With our setup, you can set all positions for the otic vesicle of each zebrafish including z levels (i.e. range and number of z slices), and then do the whole image acquisition in an automated fashion in one run. However, automated moving from fish to fish means that the microscope is moving such that the zebrafish need to be stabilized in their position. In E3+tricaine, the zebrafish are not stabilized at all, and even small vibrations let alone moving the well plate can move them during image acquisition. Thus, embedding them in LM agarose for imaging is essential (to achieve high-quality images) and faster for larger sample sizes.

**We now state in the caption of Figure 1** that we used widefield instead of confocal microscopy to allow for automated multi-sample imaging.

3) In the section "Time for infections, imaging, and analysis highly depends on the number of treatments and zebrafish injected," it seems a bit approximate, redundant, and inconsistent. Rather than having a dedicated paragraph, if the Authors really want to indicate the timing of the protocols, I would include them in the text as they explain them.

3) We agree that the timings given are approximate since they highly depend on how much practice a researcher had with this system. However, we disagree that our time indications are redundant and inconsistent. Since this is a protocol-based article (“PLOS ONE *Lab Protocols*”), an approximate time frame (per experimental unit) will be helpful for any researcher who wants to use our protocols so that (s)he can plan ahead. We think the **location of this paragraph might not be ideal** and thus **moved it** to the end of the Materials & Methods section (see lines 203-220). In addition, at the end of that paragraph, **we now point out that time estimations represent indications** that may vary in response to the experimenter’s training status and experience.

4) The Authors incubate the embryos with a 14-hour light and 10-hour dark cycle?? Please clarify this

point as usually embryos are kept in dark in the incubator.

4) In fact, both options are commonly used by researchers. To simulate a natural day-night rhythm, for example, Li & Hu (2012, Journal of Genetics and Genomics), Wehmas et al. (2016, Zebrafish), Tuschl et al. (2022, Disease Models & Mechanisms), and Zhang et al. (2024, Royal Society of Chemistry) maintain zebrafish embryos under a 14-h light and 10-h dark cycle. **We have now added both options in the protocol** (see protocol A part 3 step 40) and in the caption of Figure 1.

5) Survival experiments (from which they derive Kaplan-Meier graphs) are only done in biological duplicate, not triplicate.

5) This is correct. However, it is important to note that our manuscript is a “Lab Protocols” article. The aim of such an article is to provide step-by-step protocols and not to derive relevant biological conclusions from experimental data. For this reason, we executed our experiments in duplicates as it is sufficient to show that our infection protocol works and that results are consistent and reproducible. Furthermore, our results are robust because we have a high number of individual zebrafish per treatment with N = 37-53. **We now show the Ns for each treatment in detail** in the respective figure captions (Figures 2 and S1).

6) There is no correspondence between the fluorescence values the Authors obtain from the analysis

using their script on ImageJ and the survival data (during co-infections). For example, P. aeruginosa is always more lethal in terms of survival but consistently shows lower fluorescence compared to Klebsiella and Acinetobacter baumanii. How do they explain this?

6) Thank you for this important observation. Before we address your question, we wish to stress once more that this is a methods paper (hence, PLOS ONE *Lab Protocols*). The primary aim is to describe new step-by-step protocols and not to deliver biological insights or to test specific hypotheses. Indeed, *P. aeruginosa* is more lethal for the zebrafish larvae while showing lower fluorescence than the other two species. There are two potential reasons for this. First, *P. aeruginosa* is known for being a highly virulent and lethal pathogen, meaning that lower pathogen loads can lead to higher host mortality. We demonstrated this effect in one of our previous studies using *Galleria mellonella* larvae as a host organism (Schmitz et al. 2023, Proceedings of the Royal Society B). Second, two fluorophores might differ in their signal intensity. When investigating a biological phenomenon, it is therefore advisable to repeat experiments with swapped fluorophores (e.g., *P. aeruginosa* and *K. pneumoniae* each tagged with mCherry and GFP). We did not do this here because our study is focusing on the technical and not biological aspects of co-infections.

7) The amount of CFU injected for the different bacterial strains are higher compared to those used in similar model of infection in zebrafish. A dose dependent assay to justify the doses is necessary or a discuss possible motivation (i.e. the injection in the otic vescisle requires higher bacterial load to induce inflammation in comparison to other districts commonly used in zebrafish embryo/larvae?).

7) Thank you for this comment. Since this is a “Lab Protocols” paper, we picked specific injection doses to demonstrate that the protocols work and not to infer any biological implications of dose responses. Nonetheless, we conducted a dose-dependent assay for *P. aeruginosa* and show the results in Fig. 2A. Moreover, **we now state in our discussion that doses required for infections may vary across organs** (lines 262-265).

8) In the limitations (last paragraph), the Authors mentioned they cannot set the CFU/embryos injected properly. There is a great variability from experiment to experiment. Even though they mention discarding experiments with too much CFU/embryo variability, this remains a significant limitation because if the initial inoculum is incorrect, it can alter the development of bacterial co-infection (the ratio between the two species). Several works properly described different ways to calculate precisely the injected CFU.

8) Calculating the infected CFU precisely is not the issue. The issue is that even with precisely prepared bacterial solutions, there is variation in how many bacteria end up in the zebrafish embryo. This variation is typically not reported in papers. Here, we measured and reported it to sensitize experimenters. Since our work is a method paper, we regard it as our duty to discuss this source of variation. Pointing out this difficulty will be helpful for researchers aspiring to work on zebrafish infections. In addition, measuring this variation is also useful as it can be factored into statistical models. Importantly, we also stress that practicing infections lowers this variation in our manuscript (lines 365-367).

9) I understand the rationale is to study the interaction in terms of local infective capacity (in an organ) between two bacteria belonging to different species or genera during a co-infection. However, the only output the Authors extract from the protocol is the fluorescence value of each bacterium and the overlapped signal. They do not experimentally explain or at least discuss how they connect this single output (along with embryo survival) to a greater fitness of the bacterium in a co-infection. The model is made more robust and appealing by the fact that there is interaction even with the innate immunity in that district.

9) We agree that this model organism is particularly appealing because it allows examining interactions between pathogens and pathogens and host innate immunity. However, we wish to emphasize once more that this is a “Lab Protocols” article that offers tools to the research community. It does not address biological questions. With the tools provided, the researchers can conduct exactly the types of experiments suggested. By providing guidance on how to obtain three different outputs (namely, fluorescence values for each species, their overlapped signal, and host survival), researchers are free to adapt how they use these values tailored to their specific experiments. For example, we envision that bacterial fitness can be approximated by the area they occupy and how this area changes over time in a (co-) infection. Furthermore, one could use transgenic zebrafish lines that have macrophages or neutrophils fluorescently labeled (Benard et al. 2012, JoVE; Willis et al. 2016, Current Biology; Hajjar 2024, Scientific Reports) to connect bacterial fitness, approximated by the area occupied by bacteria, to the host’s immune response, Our tools offer all these avenues, but it is not the purpose of this paper to pursue them. We now discuss these potential avenues on lines 388ff. In addition, we had stated potential use cases of our workflow in the introduction (lines 79ff).

10) The introduction misses the mark. It fails to describe the zebrafish as a model for bacterial infection, instead delving into the broader history of zebrafish as a model organism. It's irrelevant to discuss the transparency of zebrafish embryos or their non-animal status until five days post-fertilization.

10) We disagree with the reviewer. This is a methodological paper and not an in-depth biological paper (hence our submission to PLOS ONE *Lab Protocols*). We thus focus on relevant information regarding the use of zebrafish as a model host for investigating bacterial infections. At the same time, we also describe the usefulness of the zebrafish model to study host immune responses and pathogen gene expression. Thus, we believe that our introduction offers a balanced insight into the various aspects of the zebrafish model. Moreover, our study is aimed at both researchers already working on zebrafish infections and those new to this study system. For the latter, it might indeed be relevant to learn about the non-animal status until five dpf because this might encourage them to use the zebrafish as a model host. Similarly, the transparency of zebrafish allows for examining infection dynamics in real-time in live animals, which might be another reason to switch from mice to zebrafish.

**Reviewer #2**

Review for manuscript by Schmitz DA et al titled “A new protocol for multispecies bacterial infections in zebrafish and their monitoring through automated image analysis.”

The authors described a novel method to investigate multispecies bacterial infections in a zebrafish model. Zebrafish is a very upcoming model system for infectious diseases but previous studies are only investigated monospecies infection. For that reason this study is very important and will provide a lot of opportunities to study other multispecies infections. The development of a publicly available image analysis pipeline is a very valuable addition to the study.

The manuscript is well-written, the experimental approach needs some clarifications. In the supplementary materials there are 3 very well described, detailed methodology which will be greatly appreciated for other scientists.

Thank you for the concise summary and positive feedback on our study. We appreciate that you recognize the quality of our work, but also raise concerns, which have helped us to improve our manuscript. Please find below our detailed responses to each of your comments. Whenever we state line numbers, they refer to the clean manuscript version (i.e., not the tracked changes version).

A few required corrections/ clarification:

1. In the introduction: “Nevertheless, they already have innate immunity.” Please provide references

1. Thank you for pointing this out. We have added the references on line 60.

2. It was stated that zebrafish was infected with increasing amounts P. aeruginosa in two independent experiments – why not 3 independent experiments and what were the Ns of the other mono and multi species infection experiments?

2. We repeated the experiment twice because of two reasons. First, the purpose of this article is to provide new protocols and demonstrate that they work. Two replicates seem adequate for this aim, also because we do not draw relevant biological conclusions from our data (for which biological triplicates would be necessary). Second, we used a relatively high number of fish per treatment (see list below) so that the survival curves we obtained for each experiment are consistent and reproducible. We have now included the following list in the respective figure captions.

Figure 2A: control, N = 48; low, N = 43; medium, N = 47; high, N = 45

Figure 2B: control, N = 48; *P. aeruginosa* mono, N = 45; *K. pneumoniae* mono, N = 42; mix, N = 53

Figure 2C: control, N = 48; *P. aeruginosa* mono, N = 45; *A. baumannii* mono, N = 37; mix, N = 44

Figure S1: control, N = 48; *A. baumannii* mono, N = 37; *K. pneumoniae* mono, N = 42; mix, N = 46

3. Figures are blurry, please work on them.

3. All figures have been saved with at least a 300-dpi resolution, which is recommended by the journal. Commonly, journals reduce the figure size for building a pdf file from all uploaded files included in a paper. For example, when I reviewed an article previously, there was the option to look at the original quality image separately (either in the online system directly or by downloading it there). If this still does not give well-resolved figures, please let us/the editors know so that we can find a solution.

**Reviewer #3**

In this manuscript the authors describe a new larval zebrafish inner ear model to assess infections by multiple pathogens at the same time. The have developed AI-based screens to automate the monitoring of these multiple pathogens within the ears of these fish. Imaging strategies are also described. The protocols are nicely detailed and should be easy for readers to follow.

Thank you for your valuable feedback on our manuscript. We appreciate your positive comments as well as your raising points that are unclear. Please find below our detailed responses. Whenever we state line numbers, they refer to the clean manuscript version (i.e., not the tracked changes version).

The ms is well written but could use some editing for English grammar.

Thank you for pointing this out. We have adjusted our manuscript in a few places to improve the English grammar and clarity of our text. See lines 43, 135, 208, 210ff, 382.

Specific points:

1) Figure 2: what is “survival probability?” Either they are dead or not at the specific time points so this makes no sense.

1) We understand your concern. We found this term counter-intuitive too when we started working on survival data. However, Kaplan-Meier survival curves typically display the survival probability on the y-axis. Survival probability is used because the Kaplan-Meier estimator calculates the probability of surviving beyond each time point based on the observed survival times in our study population. In other words, it is the probability that a randomly selected subject from the population will survive beyond that time point. Thus, we would like to keep “survival probability” since it is the standard in the field and routinely used in survival analysis with Kaplan-Meier survival curves.
